# Intelligent fault diagnosis of rotating machinery based on improved hybrid dilated convolution network for unbalanced samples

**DOI:** 10.1038/s41598-025-98553-4

**Published:** 2025-04-23

**Authors:** Qianqian Zhang, Caiyun Hao, Ying Wang, Kun Zhang, Haitao Yan, Zhongwei Lv, Qiuxia Fan, Chan Xu, Lei Xu, Zhuang Wen, Weihuang Liu

**Affiliations:** 1 School of Automation and Software Engineering, Shanxi University, Taiyuan, P.R. China; 2 School of Electric Power, Civil Engineering and Architecture, Shanxi University, Shanxi, China

**Keywords:** Engineering, Mechanical engineering

## Abstract

In practical industrial applications, obtaining a sufficient number fault samples for specific types of equipment fault can be challenging. As a result, there are frequently significantly fewer defect samples obtained than healthy samples, and the data samples that are obtained typically have a high noise level. To overcome these issues, this paper introduces a novel approach termed the improved hybrid dilated convolution network (HDCN) to address these limitations and enhance classification accuracy. The proposed method involves transforming the time domain vibration signal into a time-frequency domain image using short time fourier transform (STFT), enabling simultaneous extraction of frequency domain and time domain features. A multi-scale hybrid dilated convolution network is constructed to extract multiple scale fault features and identify characteristic information. Subsequently, an adaptive weight long short-term memory (LSTM) unit is designed to perform weighted fusion of multi-scale features. It can be amplifying the contribution of important features and minimizing the influence of non-relevant features. The scaled exponential linear unit (SELU) is utilized to mitigate the significant suppression of the activation function on a few class samples. Finally, the network model is simulated using the focal loss function to make it more suitable for the case where the fault samples are small and confusing. To assess the effectiveness of the suggested approach, extensive tests are carried out on simulated datasets as well as a public dataset.

## Introduction

Fault diagnosis is a critical process in various fields including engineering, manufacturing, and technology. It involves identifying and resolving abnormalities or malfunctions in systems or components. Accurate fault diagnosis is crucial for ensuring efficient, safe, and reliable operation of the system^1–5^. With the rapid advancement of technology, there is an increasing demand for effective and automated fault diagnosis methods to minimize downtime and maintenance costs while maximizing productivity^6–9^.

The intelligent fault diagnosis method for mechanical equipment involves extracting hidden fault feature from monitoring signals and automatically identifying the equipment’s health state using intelligent algorithms. This research content is currently a prominent focus in the field of fault diagnosis. Wang et al.^10^ introduced a new fault diagnosis model called Subdomain Adaptive Transfer Learning Network (SATLN) to overcome the class and domain misalignment problem in bearing fault diagnosis. Li et al.^11^ proposed a multi-receptive field graph convolutional network (MRF-GCN) for effective intelligent fault diagnosis. Zhang et al.^12^ introduced a small sample fault diagnosis method utilizing dual path convolution with attention (DCA) and Bidirectional Gated Recurrent Unit (DCA-BiGRU), which capitalizes on contemporary regularization training strategies. Additionally, Dong et al.^13^ introduced an intelligent framework for fault diagnosis, utilizing dynamic model and transfer learning specifically for detecting race faults in rolling bearings. Xie et al.^14^ proposed a novel intelligent diagnosis method based on multi-sensor fusion (MSF) and convolutional neural network (CNN). Su et al.^15^ introduced a convolutional neural network for fault diagnosis with hierarchical branches, offering three predictions from coarse to precise via distinct pathways, demonstrating robust diagnostic performance in noisy environments and varying operational conditions. In order to diagnose faults in rotating machinery components, Li et al.^16^ introduced a novel deep learning technique that uses data augmentation.

Deep learning methods within the domain of intelligent fault diagnosis of machinery has yielded promising results in identifying faults within balanced data sets. However, in practical engineering applications, the number of faulty samples is often significantly smaller than that of healthy samples, and the collected data may contain noise. This imbalance can have a detrimental effect on the performance and robustness of machine learning models. Data enhancement and resampling methods and cost-sensitive approaches to quantitatively compensate small samples are common strategies to solve the problem of data imbalance fault diagnosis, such as generative adversarial network (GAN) and synthetic minority oversampling technique (SMOTE) are classical data enhancement methods. For instance, Hao et al.^17^ proposed a novel multi-resolution fusion generative adversarial network (MFGAN) using data augmentation for the imbalanced failure diagnosis of rolling bearings. Zhou et al.^18^ proposed a novel generator and discriminator for GAN aimed at producing more distinctive fault samples through a global optimization strategy. For imbalanced defect identification, Wang et al.^19^ proposed a conditional variational auto-encoder generative adversarial network (CVAE-GAN). The cost-sensitive approach is to introduce class weights into the model training process. By giving more weight to a few classes, the model is encouraged to concentrate on learning instances of these few classes, thereby improving the overall diagnostic performance. For example, to enhance the diagnosis accuracy of unbalanced datasets, Hu et al.^20^ proposed a novel approach for diagnosing faults in imbalanced data using 1DCNN and L2-SVM. They incorporated a dynamic adjustment parameter to assign lower misclassification costs to majority class instances and introduced a new modulation factor to decrease the influence of highly discernible samples, thereby prioritizing the training of less distinguishable samples. Jia et al.^21^ proposed the deep normalized convolutional neural network (DNCNN) for imbalanced fault classification of machinery, integrating transfer learning methods to address the skewed distribution of machinery health conditions. He et al.^22^ proposed an intelligent diagnosis approach for imbalanced data utilizing Deep Cost Sensitive Convolutional Neural Network. Therefore, it is crucial to explore the influence mechanism of unbalanced data set in fault diagnosis to enhance diagnostic accuracy.

However, traditional CNNs only have a single scale convolutional kernel, which makes it difficult to extract multi-scale features with strong robustness for different types of faults, and also makes it challenging to adapt to different fault datasets. To solve this limitation, dilatational convolution can be utilized to process vibration data to obtain a large field of view without additional parameters. In order to improve the ability of convolutional feature extraction and overcome the grid effect problem of extended convolution, the hybrid dilated convolution network is used to extract multi-scale fault features of fault samples.

Meanwhile, the structure of the model plays a crucial role in the performance of deep learning techniques. Addressing the issue of deep model degradation, the residual neural network proves to be exceptionally effective^23–25^. Numerous researchers have applied the Resnet architecture to combat deep degradation in deep learning. For instance, Peng et al.^26^ proposed a novel fault diagnosis method for rotating machinery based on the deep residual neural network (DRNN) and data fusion. Additionally, Zhang et al.^27^proposed a deep residual network for mechanical fault diagnosis, incorporating selective kernel convolution along with a channel-spatial attention mechanism and feature fusion. Therefore, the principle of residual network is applied to the model, which alleviates the problem of slow convergence or unstable performance in the training process, and improves the generalization ability of the model. Furthermore, the multi-scale and data fusion method can effectually extract the fault information from the complex signal, making it suitable for the operation of mechanical equipment in a noise environment^28,29^.

To address the challenge of diagnosing diverse faults in bearing equipment under unbalanced data conditions, an improved hybrid dilated convolution network is proposed. The experimental findings indicate superior diagnostic performance of the proposed approach compared to several existing fault diagnosis techniques. The main contributions of this study are outlined as follows:

(1) A multiscale hybrid dilated convolution network module is employed to capture the fault features across various scales of the collected vibration signals, and to identify the characteristic information.

(2) The focal loss function is used to improve the applicability of network model to unbalanced samples. The SELU is utilized to mitigate the significant suppression of the activation function on a few class samples.

(3) A multiscale HDCN model with adaptive weight LSTM unit is proposed for fault classification with unbalanced data. The approach enhances fault diagnosis in intelligent machines operating under unbalanced data conditions.

(4) Experimental verification on two datasets demonstrates remarkable advantages in terms of accuracy, robustness noise, and independence from sample size.

The rest of this paper is structured as follows. Section II is mainly about the fundamental theoretical model for dilated convolution network and LSTM. In Section III, a detailed illustration of the mechanical fault diagnosis pipeline enhanced with adaptive multi-scale HDCN is provided. Section IV presents comparative experiments and analysis to demonstrate the superior performance of the proposed model. Conclusions are drawn in section V.

## Theoretical background

### Hybrid dilated convolution network

**Fig. 1:**
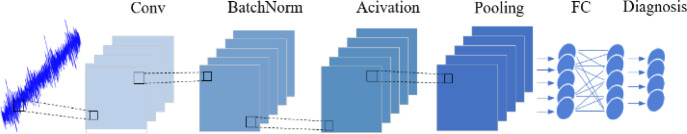
Classical convolutional network for fault diagnosis.

Classical convolutional networks are extensively employed in fault diagnosis, consisting of convolutional layer, pooling layer and activation function. Figure 1 illustrates the general CNN model for fault diagnosis.

Learning discriminative features effectively is essential for machine fault diagnosis. In order to achieve a broader field of view, convolution operations are typically repeated, which increases both training parameters and time costs. Dilated convolution can be employed to process vibration data, achieving a wide field of view without additional parameters.

However, the existing extended convolution suffers from the grid effect, that is, the features of the extended convolution are obtained through skipping. Some feature locations are frequently accessed, while others are not accessed, and resulting in the loss of some information from the original input. The utilization of hybrid dilated convolutional network offers a solution to these issues caused by resampling. It allows us to increase the receptive domain without increasing computational workload.

**Fig. 2:**
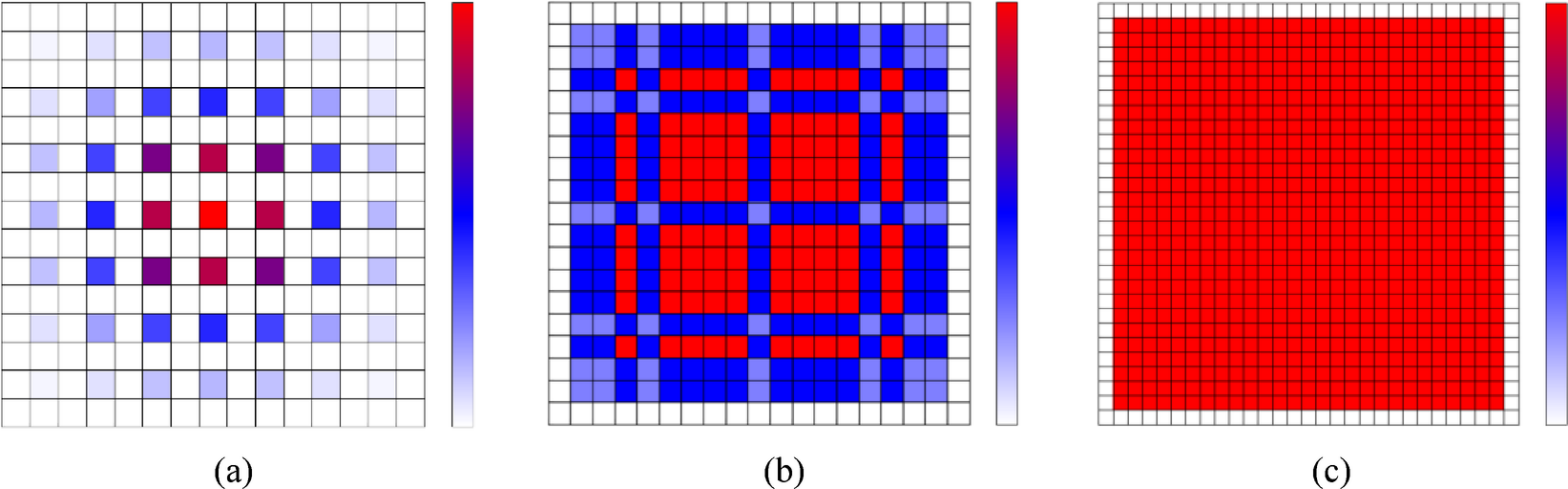
(**a**) Dilation rate = [2,2,2]; (**b**) Dilation rate = [1,2,5]; (**c**) Dilation rate = [1,3,9].

For instance, as shown in Fig. 2(a), when only 3 × 3 kernel with dilation rate is set to [2,2,2] are superimposed several times, the receptive field is limited, resulting some information from the original input is lost. As shown in Fig. 2(b), when dilation rate is set to [1,2,5], all signal features will be extracted. However, it is observed that the utilization rate of features does not reach its maximum potential. Conversely, as depicted in Fig. 2(c), when dilation rate is set to [1,3,9], the receptive field will increase and the feature utilization will be improved. Therefore, the dilation rate of [1,3,9] is chosen as the expansion parameter for the subsequent model.

### Long short-term memory

LSTM emerges as a highly advantageous solution for processing extensive data, showcasing its prowess in proficiently capturing vital information embedded in sequential data. The gate structure of LSTM is a sophisticated filter. It can skillfully expunge noise and thereby bolstering the robustness of features against disruptive elements. The foundational processing units within LSTM are denoted as “cells”, each of which is endowed with a diverse array of gates meticulously crafted to orchestrate and regulate the fluidity of information throughout sequences of varying lengths.

LSTM is able to distinguish the relevance of information between broad long-term dependencies and fleeting short-term nuances. In essence, an LSTM cell can be precisely characterized as a dynamic and adaptive information processing unit. Through its intricate gating mechanisms to manage the ebb and flow of information in sequences. Thereby a nuanced understanding of context is offered over different temporal scales. LSTM can be accurately described as:

1 $${i_t}=\sigma \left( {{W_i}\left[ {{x_t},{h_{t - 1}}} \right]+{b_i}} \right)$$

2 $$\begin{array}{*{20}{c}} {{f_t}}&{=\sigma \left( {{W_f}\left[ {{x_t},{h_{t - 1}}} \right]+{b_f}} \right)} \end{array}$$

3 $${c_t}=f_{t}^{ * }{c_{t - 1}}+{i_t}\tanh \left( {{W_c}\left[ {{x_t},{h_{t - 1}}} \right]+{b_c}} \right)$$

4 $${o_t}=\sigma \left( {{W_o}\left[ {{x_t},{h_{t - 1}}} \right]+{b_o}} \right)$$

5 $${h_t}={o_t}^{ * }\tanh \left( {{c_t}} \right)$$

where ${W_i}$, ${W_f}$, ${W_c}$ and ${W_o}$ donate the weight matrixes.${b_i}$,${b_f}$, ${b_c}$and ${b_o}$represent the biases. $\sigma$denotes the sigmoid function and tanh is the hyperbolic tangent function.

## The proposed method

Aiming to elevate the effectiveness of fault diagnosis to a superior level, an innovative approach is introduced for handling unbalanced data. The subsequent sections will expound upon the intricate details of this method, offering a comprehensive understanding of its design and implementation.

### Multi-scale feature extraction

Given the intricate nature of vibration signals in the time domain and their distinct characteristics in the frequency domain, it is proposed to introduce a STFT layer. This strategic approach aims to enhance signal features, enabling the network model to effectively discern pertinent information. The output of the STFT layer is mathematically expressed as:

6 $$S(\omega ,\tau )=\int_{{ - \infty }}^{\infty } {f(t){{\text{g}}^ * }(t - \tau ){e^{ - j\omega t}}dt}$$

where$*$ denotes the complex conjugate symbol.${\text{g}}(t)$is the window function and$f(t)$is the signal to be analyzed. The STFT is a method of converting a time-domain signal into a time-frequency domain image, through which the frequency domain and time domain features of the signal can be extracted simultaneously, helping the network model to recognize relevant information more effectively.

**Fig. 3:**
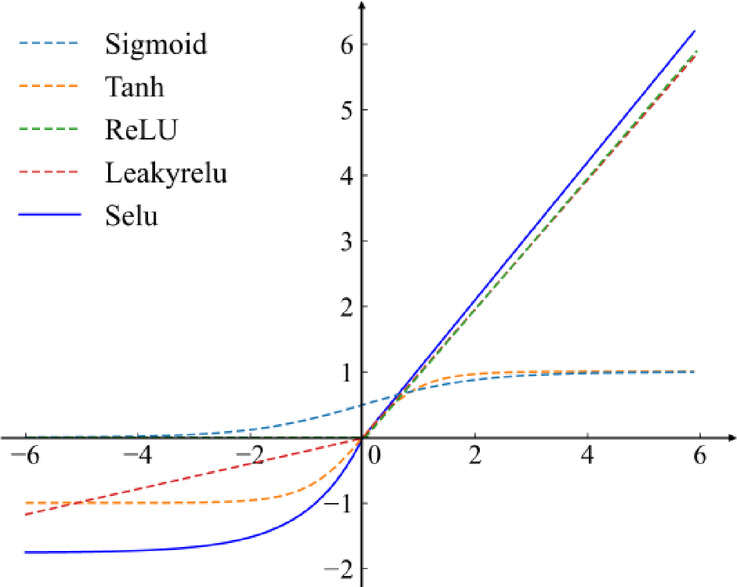
Different activation functions.

In order to improve the representation and learning ability of the network, a specific activation function is introduced between the model layers. The common related activation functions are shown in Fig. 3, where Tanh and sigmoid cause a huge vanishing gradient problem. Notably, when the input signal surpasses zero, the SELU function exhibits a curve akin to that of Relu and LeakyRelu, thereby inheriting their advantageous property of rapid convergence. SELU employs a slight negative slope for input data less than zero. This approach mitigates the vanishing gradient issue, making SELU particularly suitable for scenarios involving unbalanced data in a few class samples. In light of these considerations, SELU is chosen as the activation function for the proposed model. The experimental section will delve into a detailed exploration of the performance of different activation functions within the model, providing insights into their effectiveness.

**Fig. 4:**
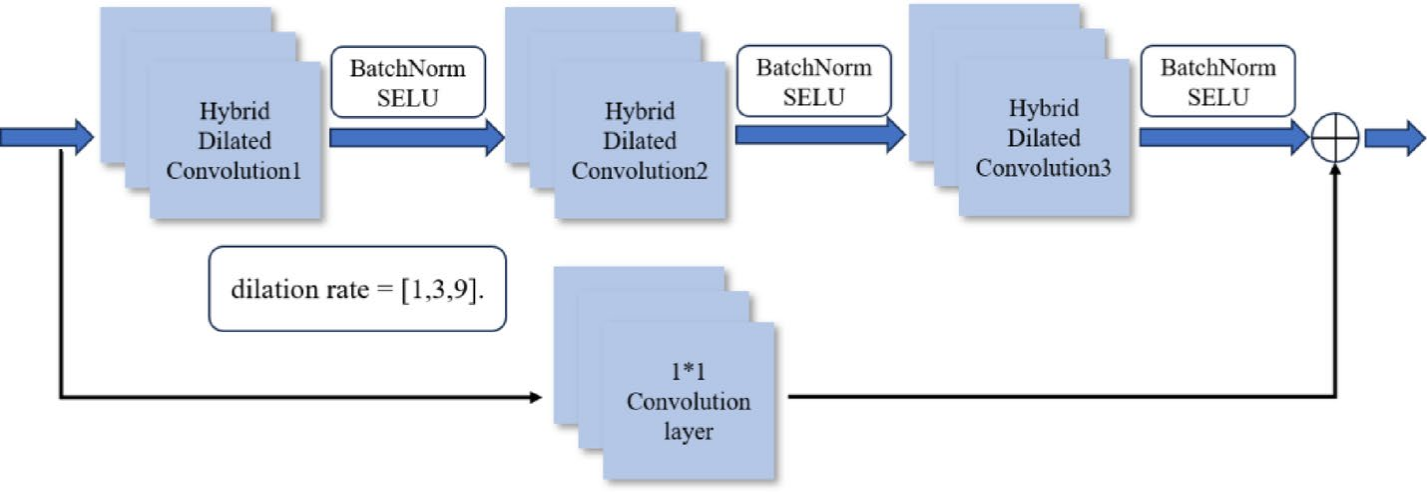
Single-channel branch block.

The multi-scale feature extraction network employs convolution kernels of varying sizes across distinct convolution units, thereby effectively capturing the nuanced differences present in fault samples with diverse convolution kernel sizes. To achieve this, we introduce a novel multi-scale CNN utilizing a hybrid dilated convolution network structure. The network comprises three parallel channels, each offering a unique perspective on the input data. Within each parallel channel, three dilated convolution units are stacked, each featuring a distinct dilation rate. Each unit consists of a convolution layer, a batch normalization layer, and an activation function. The configuration of a single-channel branch block is shown in Fig. 4. This inclusion enhances the network’s nonlinear characterization capabilities during training, addressing the challenge of deep model degradation. The utilization of 1 × 1 convolution layer serves as an essential element in refining the ability of the model to capture intricate features and maintain robust performance throughout the training process.

### Adaptive weight LSTM unit

**Fig. 5:**
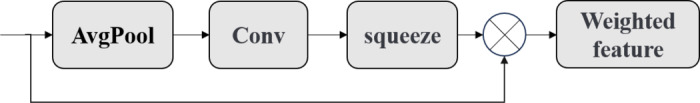
Adaptive weight unit.

The adaptive weight unit plays a pivotal role in integrating multi-scale features, intensifying the influence of crucial features, and mitigating the impact of non-correlated features. This strategic integration enhances the sensitivity of the network model to fault samples while simultaneously reducing susceptibility to potentially confusing samples, as depicted in Fig. 5.

The process begins with the globally average-pooled input feature map, which undergoes one-dimensional convolution to derive the weights associated with each channel of the feature map. These normalized weights are then multiplied channel by the original input feature, producing a weighted feature map. Subsequently, the weighted feature values are fed into an LSTM network. The adaptive weight unit, in conjunction with LSTM, is meticulously designed to accentuate the contribution of pivotal features and concurrently suppress the influence of non-relevant features. This concerted approach significantly enhances the ability of the network to discern important patterns within the input data, thereby improving its sensitivity to fault samples and fortifying its resistance to potential confounding elements.

### Fault classification

In order to mitigate the impact of varying parameter magnitudes and enhance the efficiency and accuracy of machine learning algorithms, it is often imperative to normalize the data. This practice is particularly crucial in the context of fault diagnosis to prevent a degradation in algorithm performance.

In traditional convolutional networks, the final layer typically consists of a fully connected layer, which, however, introduces a multitude of training parameters. To address this issue, a common approach in fault diagnosis literature involves substituting the conventional fully connected layer with a global average pooling layer. This substitution aims to streamline the network architecture, thereby reducing the risk of overfitting and improving the overall efficiency of training. The mathematical expression for the global average pooling layer is articulated as follows:

7 $$q\left( {{z_j}} \right)=\frac{{{e^{{z_j}}}}}{{\mathop \sum \nolimits_{k}^{{10}} {e^{{z_k}}}}}$$

When the training dataset exhibits the characteristic of imbalance, classifiers often incline toward predicting the majority class while neglecting the minority class. This tendency can lead to a decline in model performance and an incapacity to precisely predict categories associated with fewer instances of failures. The focal loss function can effectively address model performance problems caused by data imbalance.

8 $$FL\left( {{p_t}} \right)= - {\alpha _t}{\left( {1 - {p_t}} \right)^\gamma }\log \left( {{p_t}} \right)$$

9 $${p_t}=\left\{ {\begin{array}{*{20}{c}} p&{{\text{ }}if{\text{ }}y=1} \\ {1 - p}&{{\text{ }}otherwise{\text{ }}} \end{array}} \right.$$

where the ${p_t}$ is the predicted probability that the observation sample belongs to category *c*. ${\alpha _t}$ can suppress the quantity imbalance of positive and negative samples, and $\gamma$ can control the quantity imbalance of simple samples and difficult to distinguish samples.

### Diagnostic flow of the proposed method

**Fig. 6:**
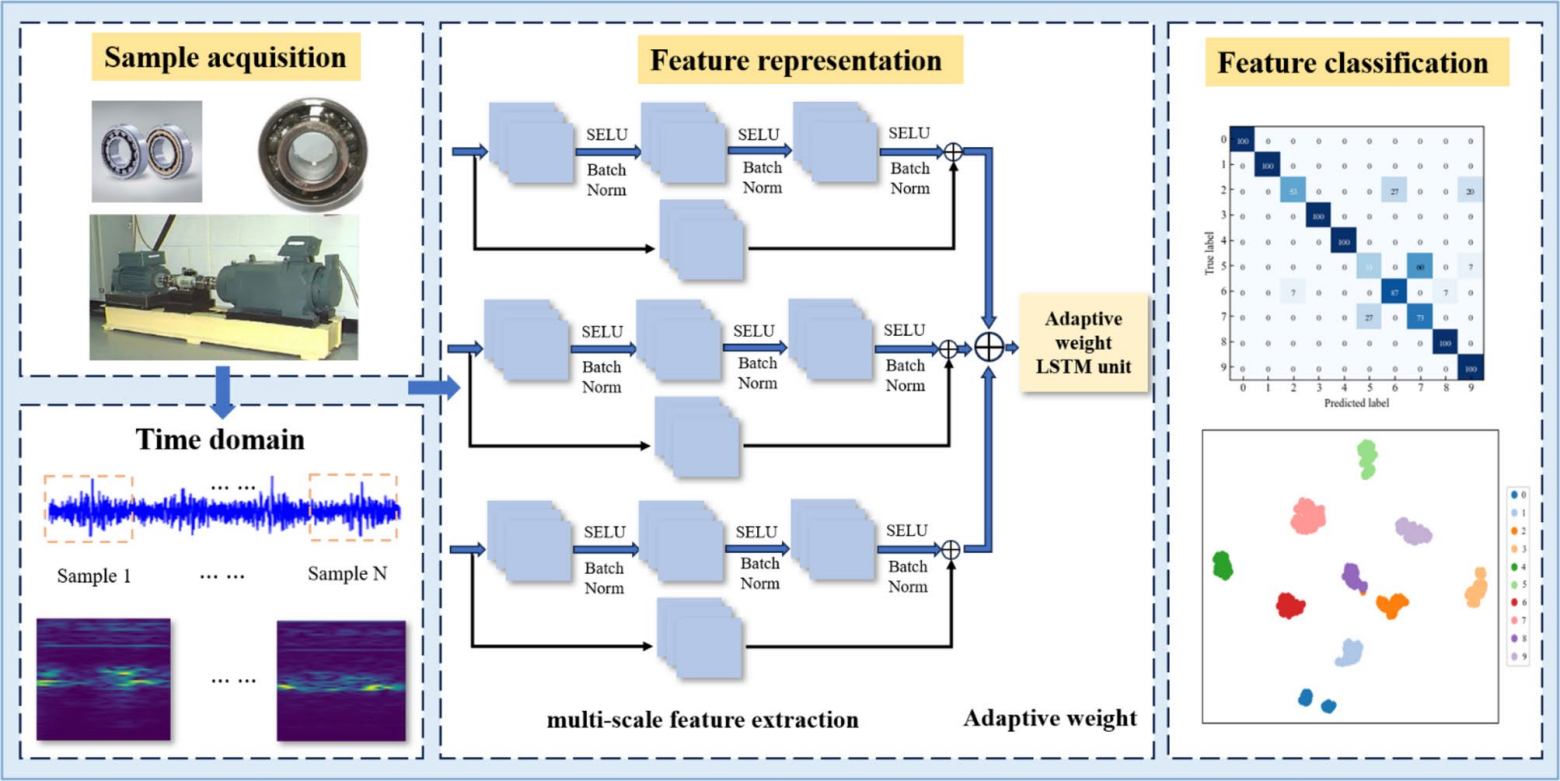
Overall framework of the proposed method.

The method presented in this study is implemented for bearing fault diagnosis of unbalanced datasets. The procedural framework of the proposed approach is visually depicted in Fig. 6, and the related parameters are shown in Table 1. The primary processes can be succinctly summarized as follows.

#### Step 1

Data preprocessing. The collected vibration signal is converted into time-frequency graphs by STFT, and then normalized.

#### Step 2

Feature extraction and fusion. The data set samples are divided into training sets and test sets, and a multi-scale and multi-channel convolutional network model is constructed. In the training process, Focal loss is used to train the method with unbalanced training samples.

#### Step 3

Fault classification. Test data sets are used for fault diagnosis, and multiple evaluation indexes are used to evaluate the diagnostic performance of the proposed model.

**Table 1:** The structure parameters of the proposed method.

Type		Dilation_rate	Kernel	Activation	Parameter
branch block_1	Conv1_1	[1,3,9]	3	SELU	96
	Conv1_2		3	SELU	1200
	Conv1_2		3	SELU	4704
	short_cut1		1	/	64
branch block_2	Conv2_1	[1,3,9]	9	SELU	672
	Conv2_2		9	SELU	10,416
	Conv2_2		9	SELU	41,568
	short_cut2		1	/	64
branch block_3	Conv3_1	[1,3,9]	17	SELU	2336
	Conv3_2		17	SELU	37,040
	Conv3_2		17	SELU	148,064
	short_cut3		1	/	64
adaptive_lstm	/		/	SELU	214,803
GAP	/		/	/	/
FC	/		/	/	10,250
					Total: 471,341

## Discussion

Experiments were conducted on two datasets to validate the effectiveness of the proposed method. All models were trained for 100 epochs, utilizing an initial learning rate of 0.0001 and the Adam optimizer. To ensure consistency, all experiments were conducted using the same random seed. Test sample accuracy values are the average results of five experimental runs, serving as the conclusive experimental outcome. The primary framework was implemented using Python. In order to facilitate fair comparison, the open-source models in^30^, including ResNet18, CNN, AlexNet, LeNet, bi-directional long short-term memory (BiLSTM) and Dilated_CNN model without adaptive weight LSTM unit, are used as comparison models in this study.

The accuracy, precision and recall rate are utilized for quantitatively assessing of the diagnostic accuracy of the above-mentioned intelligent diagnosis model. The three metrics are defined as follows:

10 $${\text{Accuracy}}=\frac{{TP+TN}}{{TP+FP+TN+FN}}$$

11 $${\text{Precision}}=\frac{{TP}}{{TP+FP}}$$

12 $${\text{Recall}}=\frac{{TP}}{{TP+FN}}$$

where TP indicates True Positive. FP indicates False Positive. FN indicates False Negative. Precision measures the proportion of true positive predictions among all positive predictions, accuracy measures the overall correctness of predictions, and recall measures the proportion of actual positives that were correctly identified as such.

### Case 1: CWRU bearing dataset

#### Data description

**Fig. 7:**
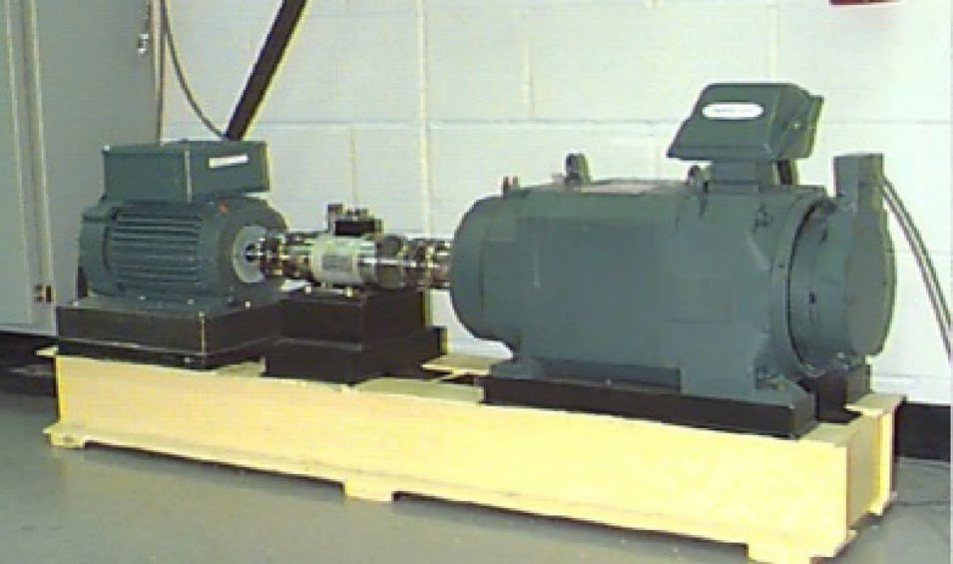
Experimental platform used by CWRU.

**Fig. 8:**
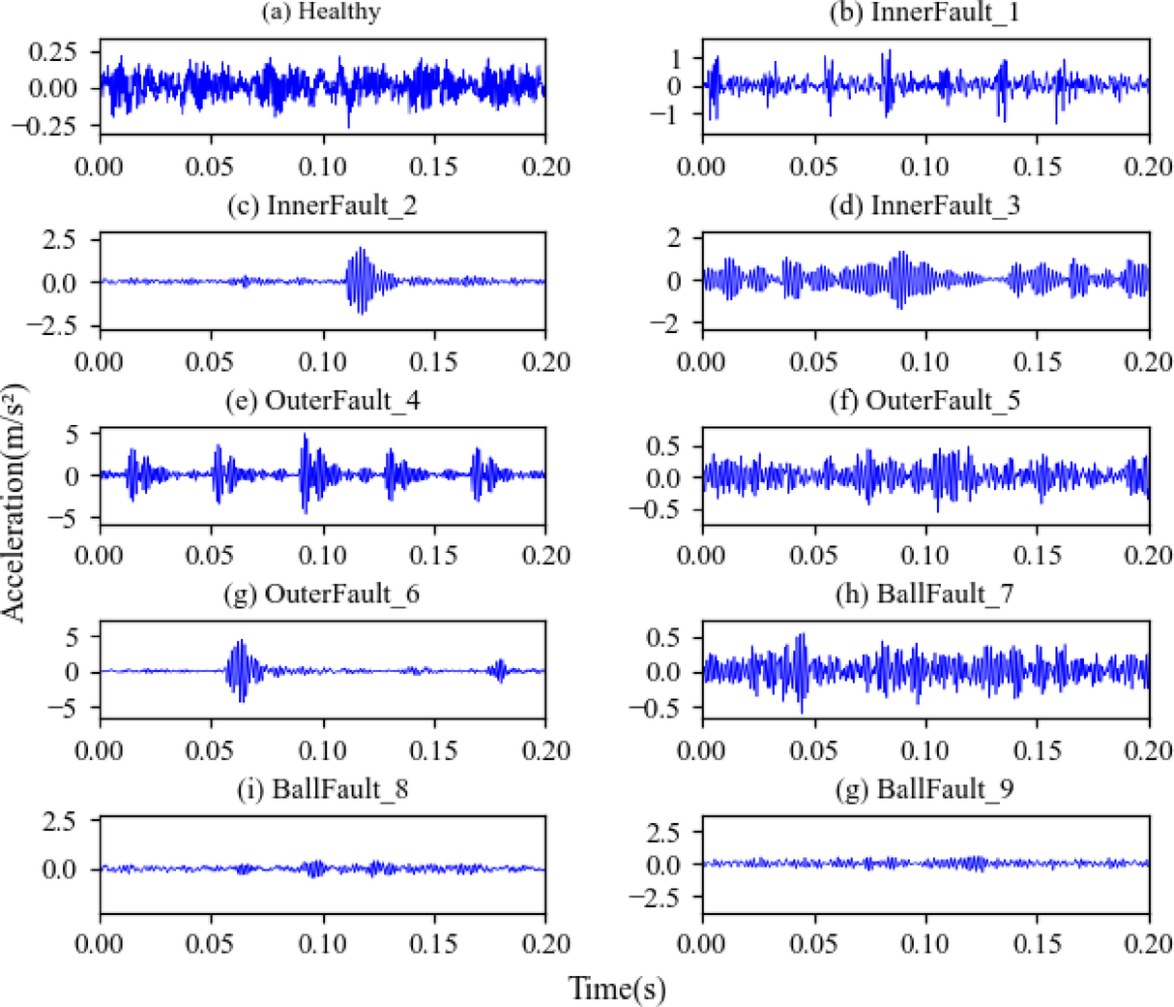
Time-domain diagram of CWRU.

The public dataset from Case Western Reserve University (CWRU) is used to assess the diagnostic validity and generalization capability of the proposed approach, as depicted in Fig. 7^31^. The dataset is a 10-classification task. The data set is constructed with data from load 4 in 48 drive end accelerometer data state. In practical mechanical operations, obtaining fault samples is more challenging compared to collecting normal samples. Therefore, the number of fault samples in dataset A is reduced to form datasets B and datasets C, which are used to simulate unbalanced data sets. Table 2 shows the results of the dataset production. Its time-domain diagram is depicted in Fig. 8.

#### The discussion of parameters

The batch size serves as a critical hyperparameter in the training of deep learning models, influencing both the training process and model performance. Smaller batch sizes contribute to enhanced gradient stability, mitigating issues such as gradient explosions or vanishing gradients during training. Conversely, larger batches can expedite training time per epoch but may compromise generalization. Striking a balance between these considerations is essential.

**Table 2:** The description of class labels of CWRU.

Fault Location	None	Inner Fault			Outer Fault			Ball Fault		
Fault Diameter	0	7	14	21	7	14	21	7	14	21
Class label	0	1	2	3	4	5	6	7	8	9
Dataset A	500	500	500	500	500	500	500	500	500	500
Dataset B	500	50	50	50	50	50	50	50	50	50
Dataset C	500	40	40	40	30	30	30	10	10	10

Utilizing dataset B, Table 3 illustrates the impact of variations in batch size and the corresponding outcomes of these variations. As we can see, the training results are different for different batch sizes, and it is clear that the diagnostic accuracy and accuracy are higher for batch sizes 16 and 32, but the time spent on batch 32 is less. Consequently, a batch size of 32 is chosen as the optimal parameter for model training.

**Table 3:** Training result under different batch size.

Batch size	Avg_acc(%)	Precision(%)	Time/s
16	99.719 ± 0.294	99.745 ± 0.261	488.136 ± 1.181
**32**	**99.860 ± 0.192**	**99.868 ± 0.180**	**475.099 ± 0.463**
64	99.368 ± 0.675	99.445 ± 0.570	472.352 ± 2.423
80	99.509 ± 0.400	99.588 ± 0.297	473.007 ± 0.801
100	99.789 ± 0.314	99.803 ± 0.294	473.227 ± 1.236

Table 4 shows the performance results of the diagnostic model with different window lengths. It can be seen that the best model structure is obtained when the window length is 256.

**Table 4:** Training result under different window length.

Window length	Avg_acc(%)	Precision(%)
**256**	**99.860 ± 0.192**	**99.868 ± 0.180**
300	99.649 ± 0.430	99.671 ± 0.403
512	99.649 ± 0.248	99.671 ± 0.233
1024	98.737 ± 0.470	98.813 ± 0.462
2048	99.017 ± 0.157	99.098 ± 0.139

Some activation functions have been extensively applied, but their potential enhancements in fault diagnosis methods have not been thoroughly explored. The comparison results for different activation functions in dataset B are shown in Table 5. For the unbalance of dataset B, the SELU activation function achieves higher precision and accuracy than other activation functions. However, the SELU method requires more training time than other methods. This extended training time is attributed to SELU enlarging the data range and its emphasis on a small number of fault samples, leading to increased memory occupation during training.

**Table 5:** The comparison results under different activation functions.

Activation function	Accuracy	Precision	Time/s
**SELU**	**99.860 ± 0.192**	**99.868 ± 0.180**	**475.099 ± 0.463**
Relu	99.649 ± 0.250	99.679 ± 0.220	469.134 ± 1.890
Sigmoid	99.368 ± 0.458	99.414 ± 0.431	474.492 ± 0.967
Tanh	99.789 ± 0.314	99.794 ± 0.312	470.540 ± 0.517
Leakyrelu	99.667 ± 0.249	99.679 ± 0.219	473.050 ± 1.314

#### Precision analysis

**Fig. 9:**
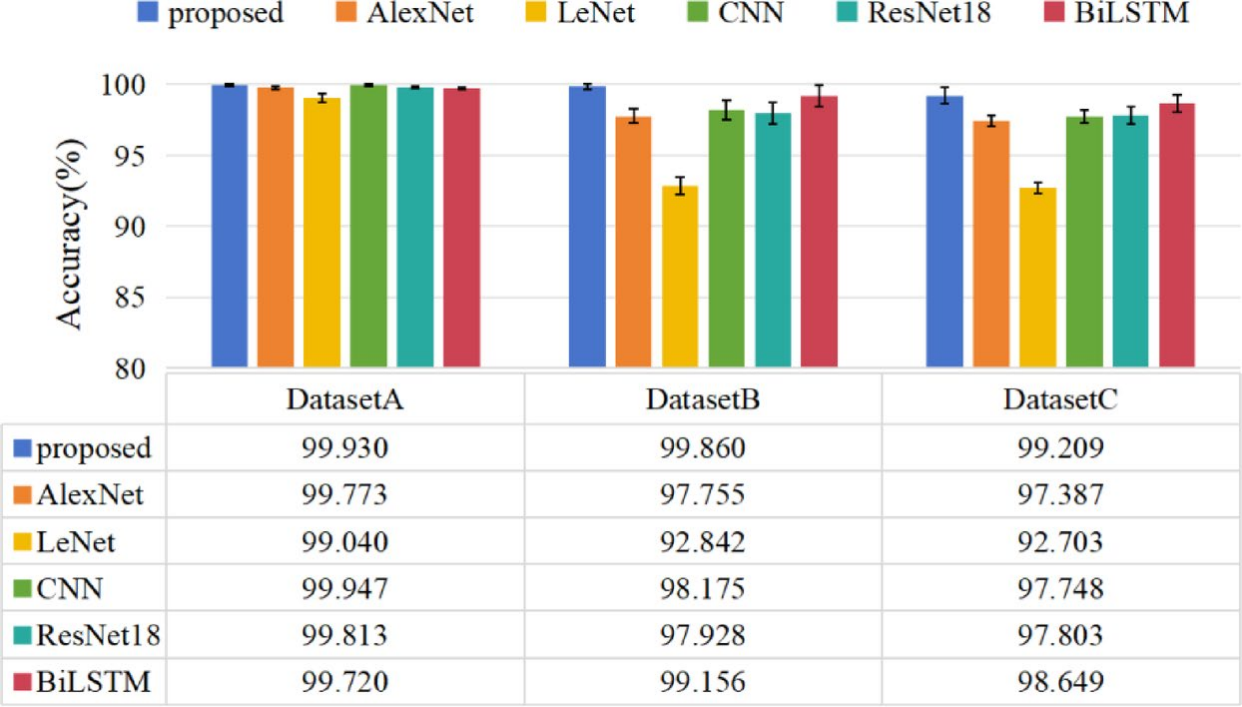
The diagnosis accuracy for each comparative method in Case 1.

 Each experiment in the following studies was conducted five times to minimize the random error with consistent training samples and hyper-parameters, with diagnostic results depicted in Fig. 9. The results are analyzed as follows: (1) The proposed method demonstrates excellent stability. Owing to the balanced characteristics of dataset A, its diagnostic accuracy surpasses that of the imbalanced datasets B and C, achieving an impressive 99.930%. Furthermore, AlexNet, LeNet, CNN, and ResNet18 all attained accuracies exceeding 99%. Notably, the proposed method showcases minimal bias, with a deviation as low as 0.047%. (2) The model achieved overall recognition accuracies of 99.860% and 99.209% on the two imbalanced datasets, respectively. The experimental results indicate that the enhanced model exhibits increased precision for minority class samples in diagnosing faults within the CWRU bearing dataset.

This improvement can be attributed to the multi-scale Hybrid Dilated Convolution network, which captures fault features at various scales from the collected vibration signals and extracts feature values. Additionally, the Focal Loss function amplifies the proportional losses for small fault samples and easily confused samples, placing greater emphasis on these instances. As a result, it enhances the diagnostic accuracy of the model when dealing with imbalanced datasets.

**Fig. 10:**
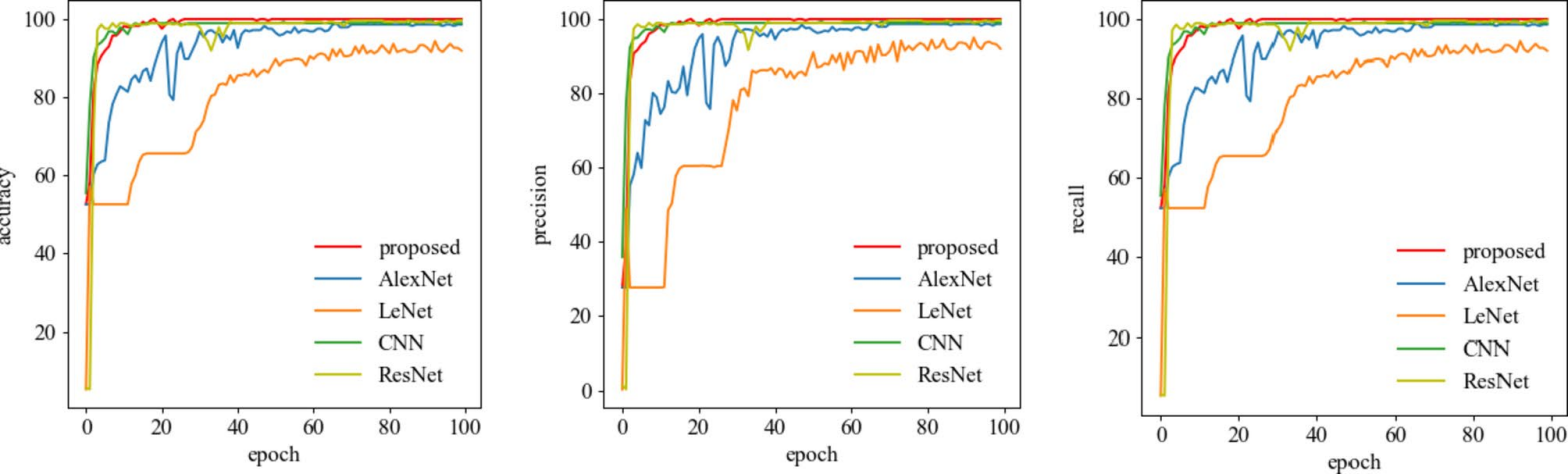
Comparison curves for the three indicators.

The accuracy, precision, and recall curves for Dataset B are illustrated in Fig. 10. This comparison further highlights that deep learning models are significantly affected by the number of training samples. Because hybrid dilated convolution network has a large acceptance domain, and the extracted multi-scale features can be distinguished effectively with strong feature information. At the same time, focal loss is used to compensate for training process, so that unbalanced fault samples can get more attention. Therefore, the method demonstrates superior diagnostic performance.

#### Anti-noise performance analysis

Machinery equipment frequently operates in noisy environments in the real world. By adding different Signal-to-Noise Ratio (SNR) noises to simulate the real environment, the robustness of the proposed method is evaluated. The expression of SNR is as follows.

13 $$SNR=10\log \left( {\frac{{{P_{signal}}}}{{{P_{noise}}}}} \right)$$

**Fig. 11:**
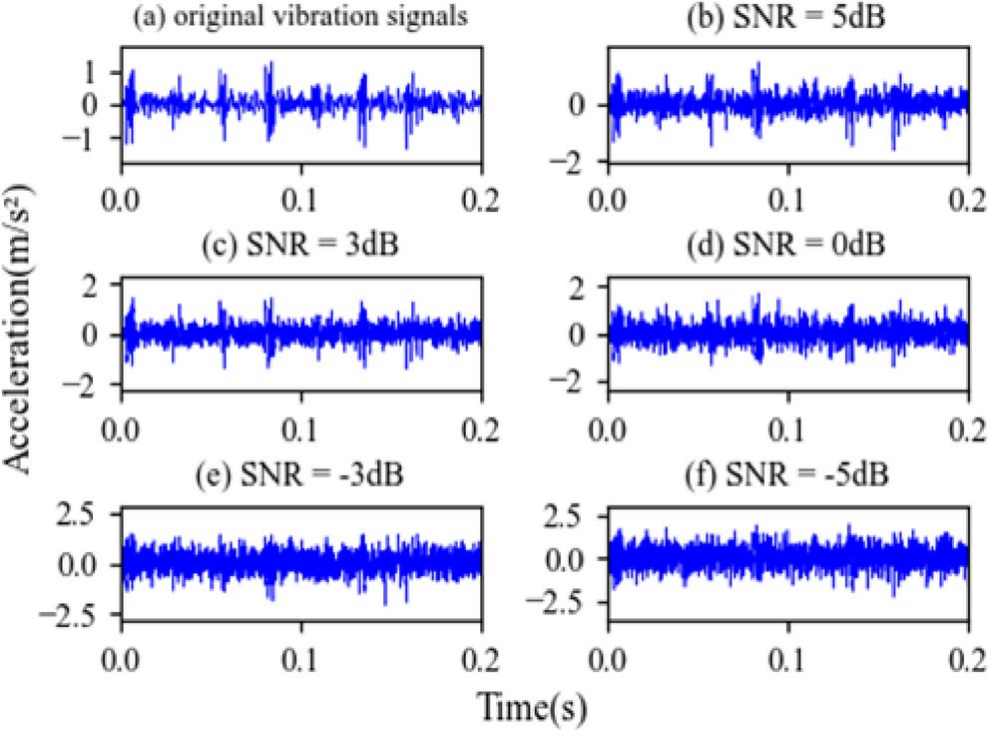
The original signal and noisy signal of Inner Fault. (**a**) Original signal; (**b**) noisy signal (SNR = 5 dB); (**c**) noisy signal (SNR = 3 dB); (**d**) noisy signal (SNR = 0 dB); (**e**) noisy signal (SNR = −3 dB); (**f**) noisy signal (SNR = −5 dB).

**Fig. 12:**
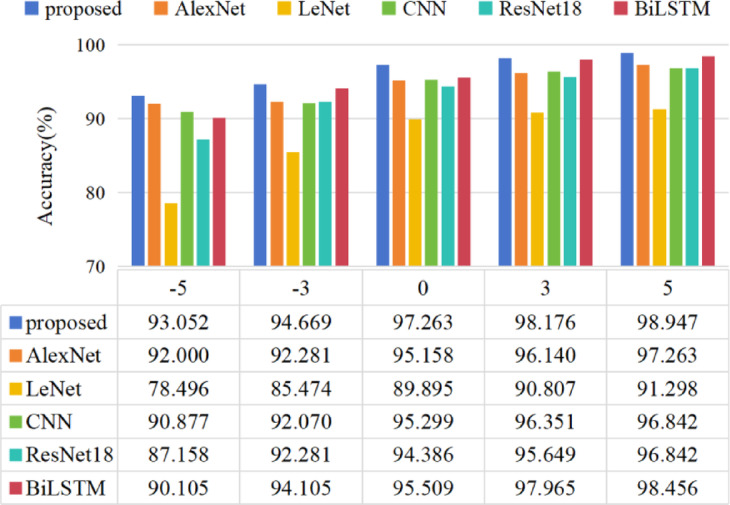
The experimental diagnosis accuracies result of CWRU under different noise level.

SNR of − 5 to 5 dB is added to the dataset B. The time-domain waveforms the original vibration signals and the noisy vibration signals of Inner Fault are shown in Fig. 11. The experimental result under different noise level is shown in Fig. 12.

Figure 12 shows the accuracy of all models after adding noise. As expected, it can be observed that the performance of models tends to decline as noise levels rise and improve as noise levels decrease. LeNet has the worst anti-noise performance. When SNR = −5 dB, LeNet only achieves an accuracy of 78.496%, while the accuracy of other models is more than 85%. The proposed method has the highest accuracy, reaching 93.052%.

### B. Case 2: Rolling bearing dataset

#### Data description

**Fig. 13:**
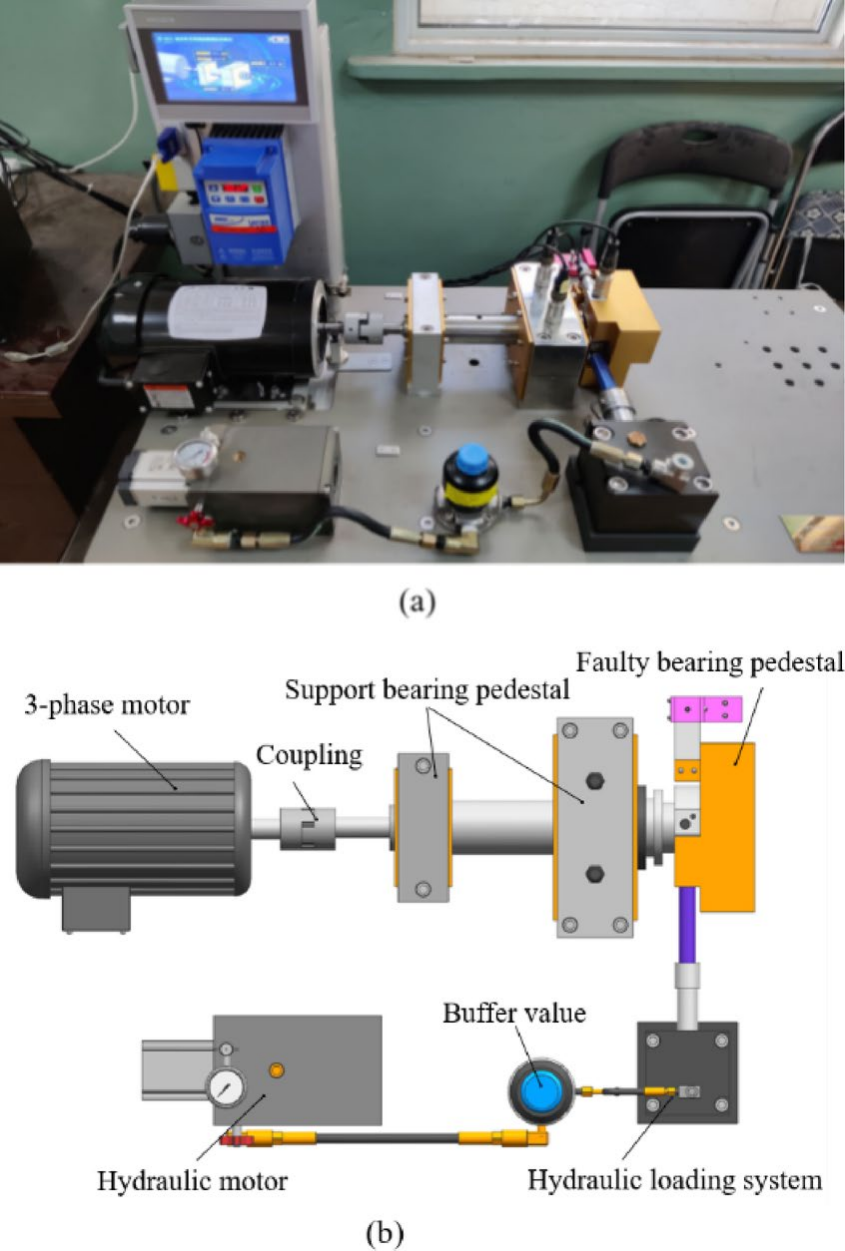
Test platform of rolling bearing: (**a**) Test platform for acquiring vibration signals^32^; (**b**) Test bench structure diagram.

Case2 dataset was collected from the test platform shown in Fig. 13, including three-phase induction motor, hydraulic loading system, normal support bearings, and faulty bearing. The acceleration sensor captured the vibration signal at a frequency of 12.8 kHz. The test rig has four states of bearing conditions: normal, inner fault, outer race fault, and ball fault. Each condition has data from three channels where the horizontal and vertical channels are in direct contact with the test bearing, and the third channel is on the bearing support box. The multi-directional signals offer complementary information, enhancing spatial-temporal resolution. Therefore, the collected signals from three directions are directly combined to construct each sample. Table 6 shows the results of the dataset production.

**Table 6:** The description of class label.

Fault Location	None	Inner Fault	Outer Fault	Ball Fault
Class label	0	1	2	3
Dataset A	500	100	100	50
Dataset B	/	100	100	50

#### Precision analysis

**Fig. 14:**
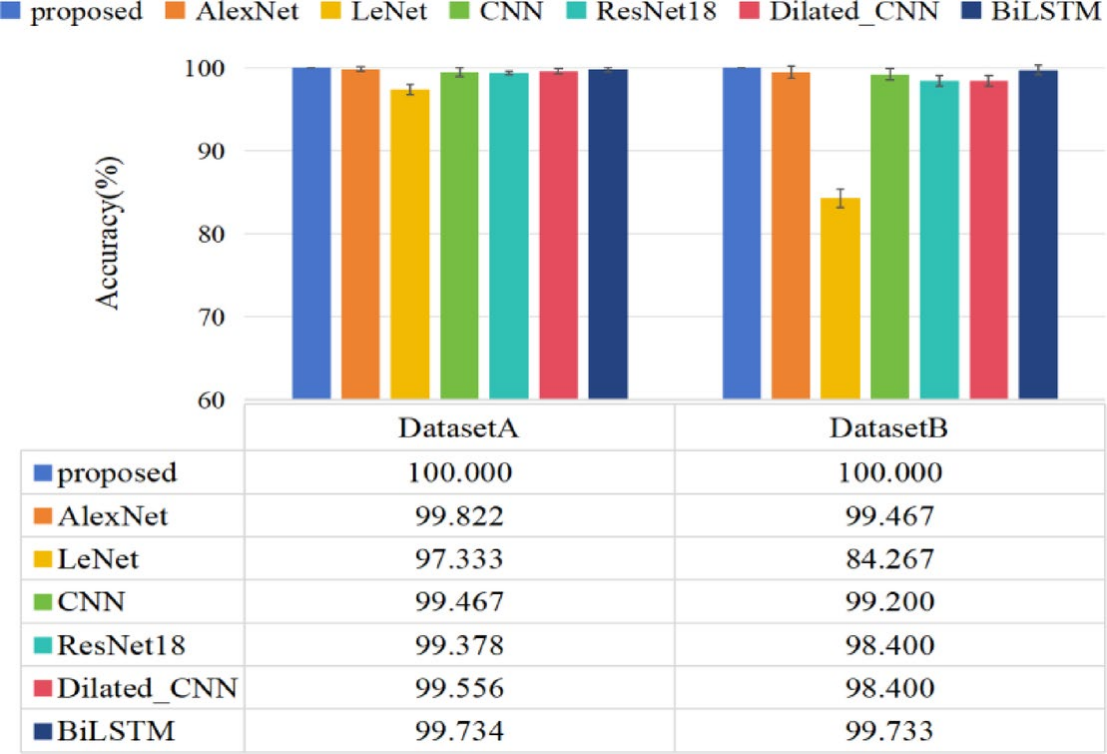
The diagnosis accuracy for each comparative method in Case 2.

Figure 14 shows the accuracy of all models. It can be seen that the proposed method achieved the highest accuracy from Figure 14 CNN and ResNet18 have also demonstrated powerful fault diagnosis capability, following only proposed model. In Case2, the proposed method has achieved accuracy of 100% on dataset A and dataset B. The accuracy of ResNet18, CNN, AlexNet, Dilated_CNN and BiLSTM is above 99% on dataset A, while the accuracy of LeNet is worse, above 95%. Dataset B contains only fault data, which significantly reduces accuracy. Because the fault data has a certain fault value, it is difficult to identify, and the proposed method can be completely identified in the case of unbalanced fault data. The proposed method demonstrated a strong classification capability.

#### Anti-noise performance analysis

**Fig. 15:**
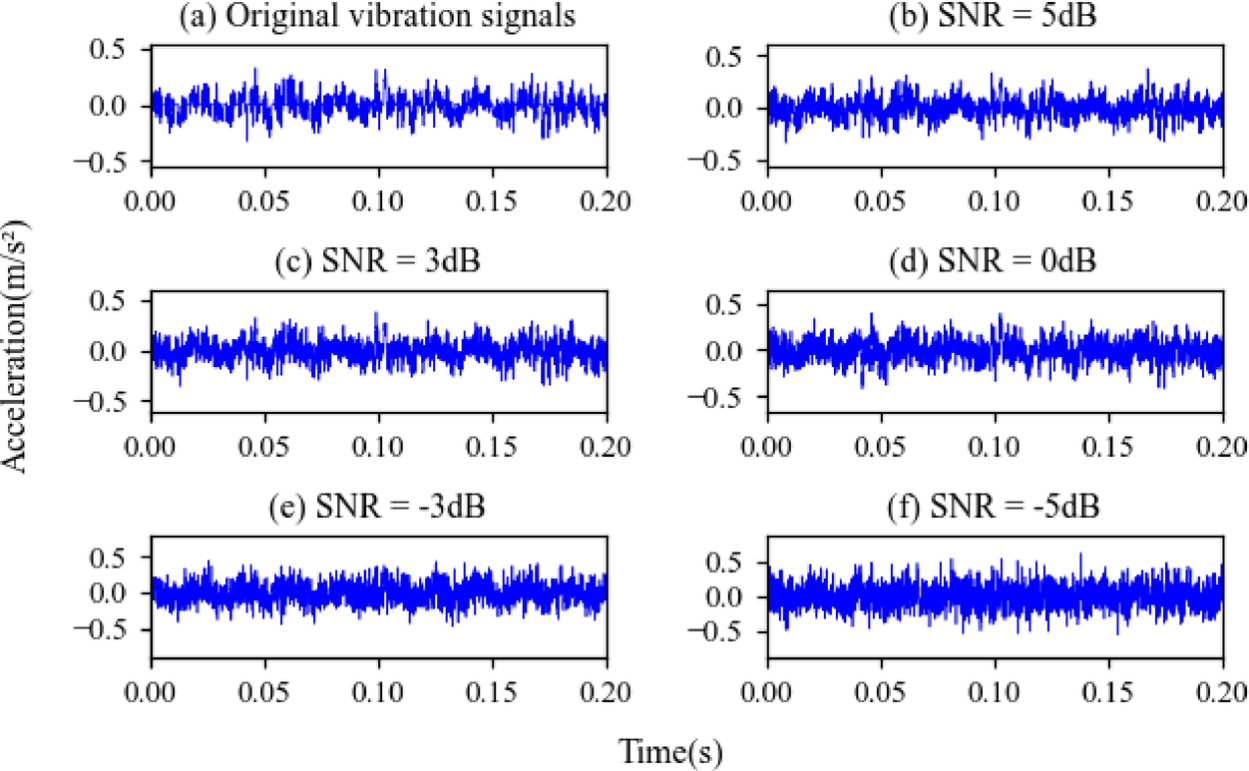
The original signal and noisy signal of Inner Fault. (**a**) original signal; (**b**) noisy signal (SNR = 5 dB); (**c**) noisy signal (SNR = 3 dB); (**d**) noisy signal (SNR = 0 dB); (**e**) noisy signal (SNR = −3 dB); (**f**) noisy signal (SNR = −5 dB).

**Fig. 16:**
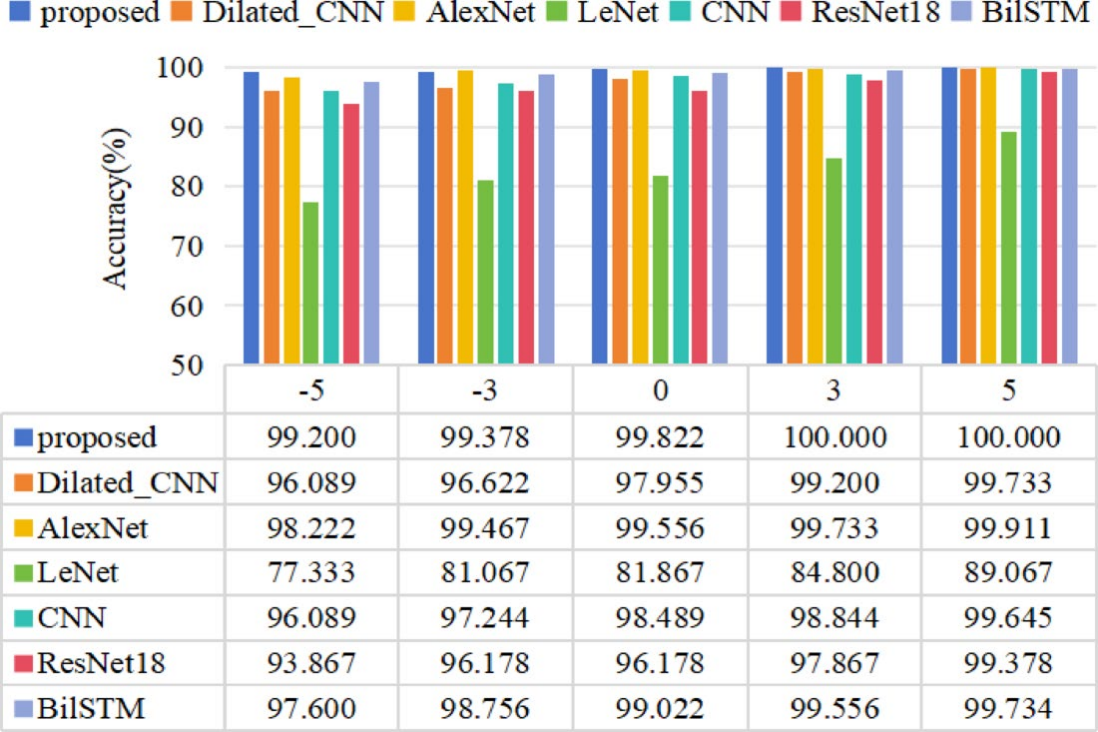
The experimental diagnosis accuracies result under different noise level.

Added the signal-to-noise ratio of −5 to 5 to Dataset A, the time domain diagram of adding noise and the accuracy of different comparison models were shown in Figs. 15 and 16 respectively. As expected, the performance of the model decreased as the noise increased, and the proposed model has higher diagnostic accuracy than other comparison models. When the signal-to-noise ratio is greater than 0, an accuracy of 100% can be achieved. The proposed method still has 99% accuracy even at a noise intensity of − 5 to 5 dB. The diagnostic accuracy of the Diated_CNN model without adaptive weight LSTM unit is obviously lower than that of our proposed method under noise interference. This further demonstrates that the adaptive weight LSTM unit possesses certain noise resistance capabilities, enhancing the robustness of the model. Although ResNet18, CNN, AlexNet, Dilated_CNN and BiLSTM achieved extremely high accuracy on the data B without added noise, the fault diagnosis accuracy decreased by more than 5% as the noise intensity increased. The LeNet model achieves the lowest accuracy under the noise background.

**Fig. 17:**
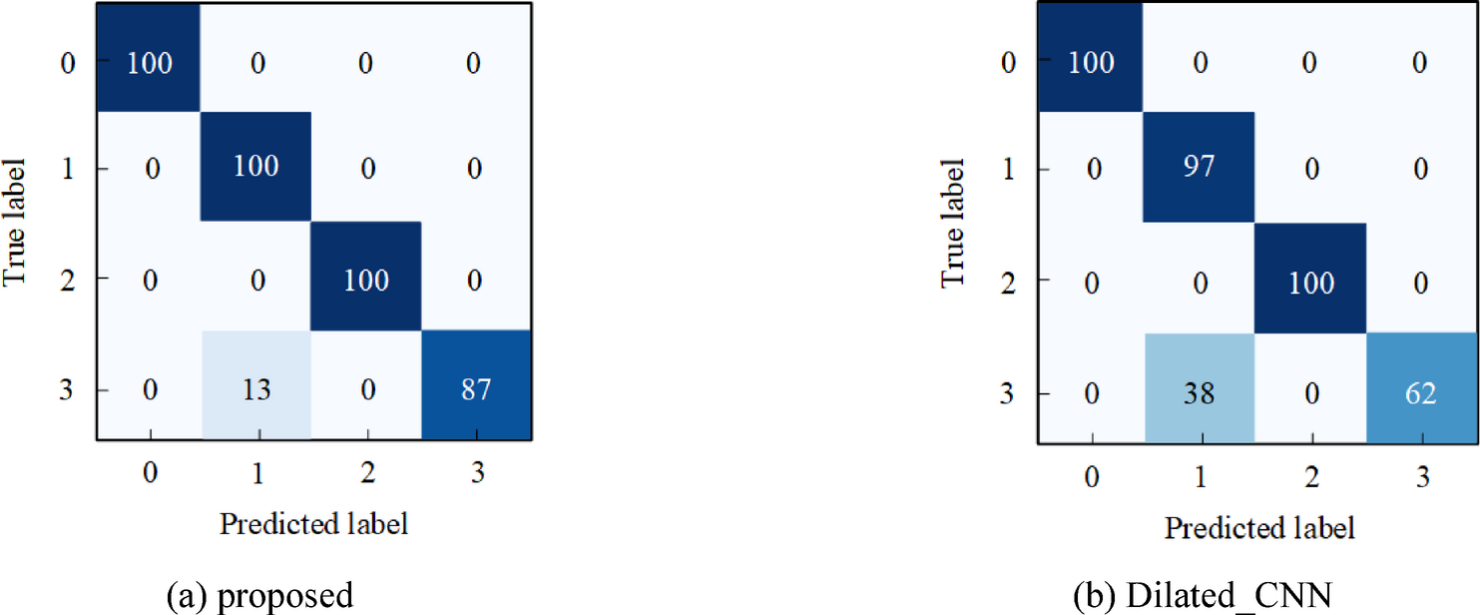
The confusion matrix with SNR of −5dB: (**a**) Proposed: 99.111%; (**b**) Dilated_CNN: 97.333%.

To illustrate the adaptability of the proposed method to various noisy fault signals, Fig. 17 presents the diagnostic confusion matrix for SNR = − 5 dB. The confusion matrix provides detailed classification results for the proposed method, where the horizontal and vertical axes denote the model predicted labels and actual data labels, respectively. As can be seen in Fig. 17, the accuracies of label 0 and label 2 have achieved 100%. For the proposed method, the accuracy of label 3 is 87%, and the rates of being misclassified as label 1 is 13%. In the Dilated_CNN model, 38% of label 3 is incorrectly classified as label 1, and 3% of label 1 is incorrectly identified as label 3. The detailed accuracy demonstrates that even the influence of noise on signal reception, the proposed method is capable of extracting robust multi-scale features from noisy signals to adapt to various fault signals.

### C. Loss function

**Fig. 18:**
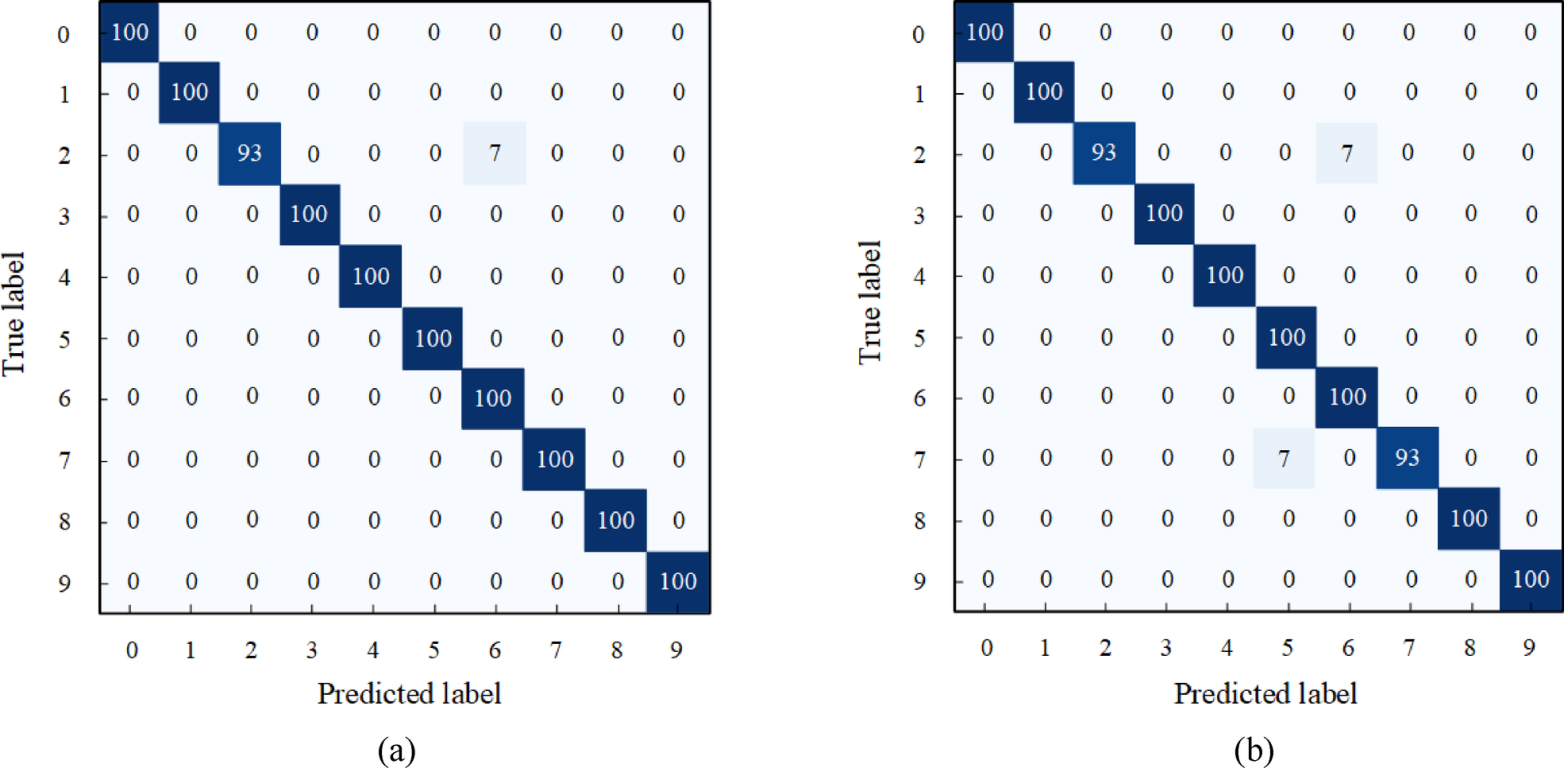
The confusion matrix: (**a**) Focal-loss; (**b**) Cross-entropy loss.

To investigate the impact of the loss function, an ablation study is conducted using dataset B from the CWRU dataset. In this experiment, the focal loss function and the cross-entropy loss function are considered. The focal loss function tends to deal with samples where there are few faulty samples and easily confused samples. Compared to cross-entropy loss, it increases its focus on minority classes by adjusting the weights of the loss function, thus improving the model’s generalization ability. Figure 18 shows the confusion matrix of the proposed model under different loss functions in the dataset B of the CWRU dataset. In the case of the cross-entropy loss function, label 2 and label 7 are incorrectly identified as label 6 and label 5. The overall accuracy of the model reached 99.298%. However, with the focal loss function, the overall accuracy of the model improved to 99.649%. Due to its increased focus on minority classes, only label 2 was misidentified as label 6. This thereby improves the overall performance of the model. The superiority of focal loss function in unbalanced data is further proved.

## Conclusion and future work

In this study, an efficiently enhanced adaptive multi-scale HDCN is proposed for intelligent fault diagnosis in rotating machinery. Firstly, vibration signals in the frequency domain are utilized to generate samples, eliminating the need of manual feature extraction. Then, the fields of view of features are enriched by using multi-scale hybrid dilated convolution. In order to improve the contribution of correlated features and reduce the interference of non-correlated features to fault diagnosis results, an adaptive weight LSTM unit is designed to achieve weighted fusion of multi-scale features. The adaptive weight LSTM unit has certain anti-noise ability and enhances the robustness of the model. Finally, the focal loss function is used to improve the applicability of network model to unbalanced samples. To assess the effectiveness of the suggested approach, extensive tests are carried out on simulated dataset as well as a public dataset.

Addressing rotating machinery fault diagnosis represents a significant and complex engineering challenge. Our future investigations will delve deeper into AI-based classification algorithms, focusing on interpretability and feature visualization aspects.

## Data Availability

The datasets generated and analyzed during this study are not publicly available due to being a data-confidential project, but are available from the corresponding authors upon reasonable request.
